# Quantitative Proteomic Analysis for High- and Low-Aflatoxin-Yield *Aspergillus flavus* Strains Isolated From Natural Environments

**DOI:** 10.3389/fmicb.2021.741875

**Published:** 2021-09-21

**Authors:** Tao Li, Zhaowei Zhang, Yu Wang, Ying Li, Jiang Zhu, Rui Hu, Yunhuang Yang, Maili Liu

**Affiliations:** ^1^State Key Laboratory of Magnetic Resonance and Atomic Molecular Physics, Key Laboratory of Magnetic Resonance in Biological Systems, Wuhan National Laboratory for Optoelectronics, National Center for Magnetic Resonance in Wuhan, Wuhan Institute of Physics and Mathematics, Innovation Academy for Precision Measurement Science and Technology, Chinese Academy of Sciences, Huazhong University of Science and Technology, Wuhan, China; ^2^University of Chinese Academy of Sciences, Beijing, China; ^3^Chinese Academy of Agricultural Sciences, Wuhan, China

**Keywords:** *Aspergillus flavus*, natural environments, quantification proteome, aflatoxin production, MRM-HR

## Abstract

The molecular mechanisms underlying aflatoxin production have been well-studied in strains of the fungus *Aspergillus flavus* (*A. flavus*) under artificial conditions. However, aflatoxin biosynthesis has rarely been studied in *A. flavus* strains isolated from field conditions with different aflatoxin-producing ability. In the present study, tandem mass tag (TMT) labeling and high-performance liquid chromatography (HPLC) coupled with tandem-mass spectrometry analysis were used for proteomic quantification in natural isolates of high- and low-aflatoxin-yield *A. flavus* strains. Additionally, findings obtained using the TMT-labeling method were validated using the high-resolution multiple reaction monitoring (MRM-HR) method. In total, 4,363 proteins were quantified, among which 1,045 proteins were differentially expressed between the high- and low-aflatoxin-yield *A. flavus* strains. Bioinformatics analysis showed that the up-regulated proteins were significantly enriched in carbon-related metabolism and the biosynthesis of secondary metabolites, whereas the down-regulated proteins were enriched in oxidative phosphorylation. Moreover, GST proteins were found to be significantly down-regulated in high-yield *A. flavus* strains; this result contradicted previous findings obtained from *A. flavus* strains grown under artificial conditions. In summary, our study provides novel insights into aflatoxin regulation in *A. flavus* under field conditions and could facilitate the development of various strategies for the effective control of aflatoxin contamination in food crops.

## Introduction

*Aspergillus flavus* is a saprotrophic and pathogenic fungus that causes disease in plants, animals, and humans and has a cosmopolitan distribution. Aflatoxins (AFs) are polyketide-derived secondary metabolites produced by several fungal species, and *Aspergillus flavus* is one of the primary producers of these compounds. Studies have demonstrated that chronic exposure to sublethal concentrations of AFs can have multiple negative health consequences, including immune suppression in humans and animals, infertility, endocrine problems, and teratogenicity related to congenital malformations and hepatocellular carcinoma (Bbosa et al., 2013). In addition, AF contamination reduces the product quality of crop plants as well as their export value, which may result in significant economic losses for countries and entities involved in commodity value chains (Wang et al., 2016). So far, many efforts have been put to study aflatoxin synthesis and pathway regulation through genetics, molecular biology and biochemistry methods because it is essential to understand the molecular mechanisms underlying AF biosynthesis to counter the increasing threat of aflatoxicosis.

In recent decades, numerous studies have shown that AF production is influenced by many abiotic factors, including water activity, temperature, and oxidative stress and biotic factors such as transcription factors or AF-related genes. Zhang et al. (2015) compared the proteomic profiles of *A. flavus* in response to different water activity levels and found that the secretion of extracellular hydrolases increased as the water activity was elevated, indicating that extracellular hydrolases are crucial for inducing AF biosynthesis. By comparing AF production at different temperatures, Wang et al. concluded that the factors affecting AF biosynthesis were not controlled by a single variable (Wang et al., 2019). Through a proteomic analysis of *A. flavus*, Fountain et al. found that AF production might contribute to oxidative stress tolerance (Fountain et al., 2018). Additionally, in their proteomic profiling study, Lv et al. found that laeA, a global regulatory factor in *A. flavus*, impairs AF biosynthesis (Medina et al., 2017; Lv et al., 2018). These results indicate that a variety of factors can induce or inhibit AF synthesis in *A. flavus*. Although these studies have provided valuable information on the mechanism underlying AF biosynthesis, most of them were performed using model strains (e.g., *A. flavus* NRRL3357, one of the most widely utilized strains for studying AF production, was the first *A. flavus* strain that was completely genome sequenced) (Amaike and Keller, 2011) under artificial experimental conditions by controlling water activity, temperature, and oxidative stress or by manipulating the expression of certain genes. Given that the physiological performance of *A. flavus* strains is greatly influenced by the environment, the analysis of natural *A. flavus* isolates with different AF production capabilities could be a promising approach for understanding AF production in real-life settings (Palkova, 2004). However, there are few studies focusing on aflatoxin biosynthesis of *A. flavus* under field conditions.

In terms of research strategies, the molecular mechanism of AF production has largely been examined using classical biological methods such as gene editing (e.g., mutation, deletion, and complementation), western blotting, and immunoprecipitation (Scherm et al., 2005; Bhatnagar et al., 2006; Tao and Chung, 2014; Ren et al., 2017; Yuan et al., 2018). Some vital enzymes and regulators (e.g., laeA, aflR, atfB, mtfA, and RsmA) involved in AF biosynthesis have been identified through these approaches (Bok and Keller, 2004; Yu et al., 2004; Bok et al., 2006; Roze et al., 2013; Zhuang et al., 2016). However, the above-mentioned methods do not allow researchers to fully examine complex protein–protein interactions (PPIs) and obtain a well-rounded understanding of the underlying mechanisms owing to their inherent low-throughput nature. High-resolution mass spectrometry (MS)-based proteomic analysis, a high-throughput method for the identification and quantification of the functional proteome, provides a systemic approach for examining PPIs (Chandramouli and Qian, 2009). Tandem mass tag (TMT) labeling is a powerful MS strategy that allows accurate proteome quantification (Pappireddi et al., 2019) and has been widely used to characterize protein profiles (Xinxin et al., 2019; Bathla et al., 2020). For example, TMT-labeling-based MS analysis was performed to compare the AF production-related proteome in *A. flavus* at different temperatures in different media. It revealed that AF synthesis is a complex process that is affected by a variety of factors such as oxidative stress, sclerotia development, G protein signaling pathways, and valine, leucine, and isoleucine degradation (Wang et al., 2019). Quantitative proteomics is thus becoming a powerful tool for studying the intricate molecular mechanism of AF biosynthesis.

In this study, we isolated five *A. flavus* strains with different AF production capabilities from natural environments. To elucidate the molecular mechanism underlying AF production, we performed novel quantification-based proteomic analysis using the TMT-labeling method and compared proteomes between high- and low-AF-yield *A. flavus* strains. This work provides a new strategy to study the underlying mechanism associated with aflatoxins biosynthesis and could promote the development of innovative strategies to control aflatoxins production under field conditions.

## Materials and Methods

### *Aspergillus flavus* Strains and Media

*Aspergillus flavus* HA, *A. flavus* HB, *A. flavus* HC, *A. flavus* LA, and *A. flavus* LB, obtained from Chinese Academy of Agricultural Sciences, were used for MS analysis in this study. The original sources of the five different *A. flavus* strains cannot be disclosed to the public owing to a signed guarantee as part of a confidentiality policy. *A. flavus* was cultured in liquid or solid PDB medium (potatoes: 5 g/L, peptone: 10 g/L, dextrose: 15 g/L, and NaCl: 5 g/L). The solid medium additionally contained agar powder (15 g/L). Conidia obtained from agar solid media were counted with a hemocytometer and inoculated into 10 mL solid PDA media at a concentration of 10^5^ conidia/mL. Subsequently, the strains were incubated at 28°C for 5 days, and the colony phenotype was recorded daily to observe the growth rate of the strains. The natural isolates were obtained from the soil of the peanut field and cultured in laboratory for biological experiments within three generations after isolation.

### Aflatoxin Production Analysis and Internal Transcribed Spacer Sequencing

For AF production assays, all strains were inoculated in PDB liquid media at a concentration of 10^5^ spores/mL. After incubation at 28°C with shaking at 180 rpm for 5 days, AF was extracted following a previously described protocol (Wacoo et al., 2014). For further analysis, the extracts were filtered (0.22 μm) and processed *via* HPLC using a Boston Boschrom ODS C18 column (4.6 Vmm × 150 Vmm, 5 Vμm) at 42°C, followed by eluted at a rate of 1 mL/min with 45% (v: v) methanol. The emission and excitation wavelength of the fluorescence detector were set as 440 and 360 nm, respectively. AFB1 (100 ppb) was used as the standard. To eliminate the random errors caused by the system, each *A. flavus* strains had three biological repeats and were mixed together for further aflatoxin production analysis. For ITS and calmodulin genes sequencing, the five *A. flavus* strains were inoculated in PDB liquid media and incubated at 37°C for 3 d. Then, their genomes were extracted through CTAB method as previously described (He et al., 2007) and then sent to Sangon Company (Shanghai, China) for ITS and calmodulin gene sequencing. The primer sequences were as follows and synthesized by Sangon Company (Shanghai, China) (Okoth et al., 2018):

ITS1F: TCCGTAGGTGAACCTGCGG;
ITS4R: TCCTCCGCTTATTGATATGC;
CF1:AGGCCGAYTCTYTGACYGA;
CF4:TTTYTGCATCATRAGYTGGAC.

### Protein Extraction, Trypsin Digestion, and HPLC Fractionation

The washed mycelia samples were first divided into two groups, the high AF group (*A. flavus* HA, *A. flavus* HB, and *A. flavus* HC) and the low AF group (*A. flavus* LA and *A. flavus* LB). The samples were ground in liquid nitrogen, and the powder was transferred to a 5-mL centrifuge tube and sonicated three times on ice using an ultrasonic processor (Scientz company, Ningbo, China) in lysis buffer (8 M urea, 2 mM EDTA, 10 mM DTT, and 1% protease inhibitor). The remaining debris was removed by centrifugation at 20,000 × *g* at 4°C for 10 min. Finally, the protein was precipitated with cold 15% TCA for 2 h at −20°C. After centrifugation at 4°C for 10 min, the supernatant was removed. The remaining precipitate was washed thrice with cold acetone. The protein was re-dissolved in buffer (8 M urea and 100 mM TEAB; pH 8.0) and the protein concentration was determined using a bicinchoninic acid protein assay kit (Tiangen, Beijing, China). For digestion, the protein solution was reduced with 10 mM DTT for 1 h at 37°C and alkylated with 20 mM IAA for 45 min at room temperature (∼25°C) in the dark. The protein sample was diluted by adding 100 mM TEAB to a urea solution with a concentration less than 2 M. Finally, trypsin (Promega, Wisconsin, United States) was added at a 1:50 trypsin-to-protein mass ratio for the first digestion, which was allowed to occur overnight, and at a 1:100 trypsin-to-protein mass ratio for another 4 h-digestion. All the experiments were conducted in three biological replicates.

After trypsin digestion, the peptides were desalted on a Strata X C18 SPE column (Phenomenex, CA, United States) and speed vacuumed for drying. They were re-suspended in 0.5 M TEAB and processed using the TMTsixplex^TM^ kit (ThermoFisher Scientific, MA, United Stated) based on manufacturer’s instructions (detailed labeling information is listed in Supplementary Table 1). Then, the individually labeled samples were pooled in 1:1:1:1:1:1 ratio (m:m) and fractionated by high pH reverse-phase HPLC using an Agilent 300Extend C18 column (5 μm, 4.6 mm × 250 mm). Briefly, peptides were eluted at a rate of 400 nL/min with a linear gradient of 2–60% acetonitrile (10 mM ammonium bicarbonate, pH 10) across 80 min. Eighty fractions were collected and then combined into 18 fractions, which were then dried using vacuum centrifuging.

### LC-MS/MS Analysis

Dried peptides were resuspended in 0.1% FA until the final concentration became 0.25 μg/μL. Then, 0.5 μg of peptides was separated using a reversed-phase pre-column (Acclaim PepMap 100, Thermo) connected to a reversed-phase analytical column (Acclaim PepMap^TM^ RSLC, Thermo). The gradient was created by increasing solvent B (0.1% FA in 98% ACN) concentration from 6 to 22% over 26 min, 22 to 35% over 8 min, and 35 to 80% over 3 min, and then maintaining it at 80% for the last 3 min. The flow rate remained constant at 400 nL/min throughout the run, which was performed on the EASY-nLC 1000 UPLC platform.

The peptides were subjected to nano spray ionization (NSI) followed by tandem mass spectrometry (MS/MS) in the Q-Exactive^TM^plus system (Thermo) coupled online to the ultra performance liquid chromatography system. Intact peptides were detected in the Orbitrap at a resolution of 70,000. Peptides were selected for MS/MS using a normalized collision energy setting of 28 and 32. Ion fragments were detected in the Orbitrap at a resolution of 17,500. A data-dependent procedure in which one MS scan was followed by 20 MS/MS scans was used for the top 20 precursors showing an ion count value above 10,000 in the MS survey scan at a 30.0-s dynamic exclusion. The electrospray voltage applied was 2.0 kV. Automatic gain control was used to prevent overfilling of the Orbitrap; 5 × 10^4^ ions were collected to generate the MS/MS spectra. For MS scans, the m/z scan range was 350 to 1,800. The fixed first mass was set to 100 m/z.

### Database Search

The resulting MS/MS data were processed using MaxQuant (Tyanova et al., 2016) with an integrated Andromeda search engine (v.1.5.2.8). Tandem mass spectra were searched against the *Aspergillus flavus* database (UniProt ID: UP000001875) on the UniProt website (https:). Trypsin/P was specified as the cleavage enzyme, and two maximum missed cleavage sites were permitted. Mass deviation was set to 20 ppm and 0.02 Da for precursor ions and fragment ions, respectively. Carbamidomethylation on Cys was specified as the fixed modification, and oxidation on Met and acetylation on the protein N-terminus were specified as variable modifications. The maximum false discovery rate (FDR) thresholds for protein, peptide, and modification site were all set to 1%. Minimum peptide length was set to 7. For quantification, TMT-sixplex was selected. Mass spectral data obtained in this study have been deposited in the ProteomeXchange repository with the dataset identifier PXD027517.

### Bioinformatics Analysis

The quantified proteins were grouped according to biological process, cellular components, and molecular function based on gene ontology (GO) annotation using the OmicsBox software (Conesa et al., 2005). GO terms, Kyoto Encyclopedia of Genes and Genomes (KEGG) pathways, and Pfam domain enrichment were analyzed using DAVID 6.8 bioinformatics resources (Jiao et al., 2012) with a corresponding *p*-value < 0.05, which was considered statistically significant. The differentially expressed proteins (DEPs) were also mapped to metabolic pathways using the KEGG pathway website^1^. For PPI network analysis, interaction data were obtained from the STRING database and visualized using Cytoscape software (version 3.6.1) (Shannon et al., 2003). Heatmaps and volcanic maps were drawn using the TBtools software (Chen et al., 2020).

### High-Resolution Multiple Reaction Monitoring Verification

The peptides were fractionated by HPLC using an Eksigent NanoLC 425 C18 column (15 cm × 0.3 mm). Briefly, peptides were eluted at a rate of 5 μL/min with a linear gradient of 5–80% acetonitrile across 85 min. MS analysis was performed on a TripleTOF 5600 LC-MS/MS System (AB SCIEX) using both the MRM-HR Workflow3,4 and Scheduled MRM-HR Workflow. The accumulation time of the time-of-flight (TOF) MS scan was 250 ms, and the scan range was set to 350–1250. The product ion scan (TOF MS/MS scan) was performed in the high-sensitivity mode, and the scan range was 100–1500. Full scan MS/MS was performed in the high-sensitivity mode with an optimized accumulation time per cycle. Collision energy was set based on rolling collision energy with a collision energy spread of 5 V. Proteins and peptides were selected for analysis, and MRM-HR workflow data was acquired and processed to determine peptide retention times. The retention time window used for most datasets was 2.5 min, although for 53 peptides, a window of 2.0 min was used. The final scheduled MRM-HR workflow acquisition methods and MultiQuant Software quantitation methods were developed simultaneously by Skyline (Pino et al., 2020). Final data processing was performed using MultiQuant 3.0.3 Software (AB SCIEX). For protein quantification, the abundance of each protein was calculated by summarizing the peak area of the corresponding peptides. The mean value of targeted peptide abundance was used to calculate the fold change (FC) for samples of the same protein.

## Results

### Natural Isolates of *A. flavus* Strains With High and Low Aflatoxin Yields

**FIGURE 1:**
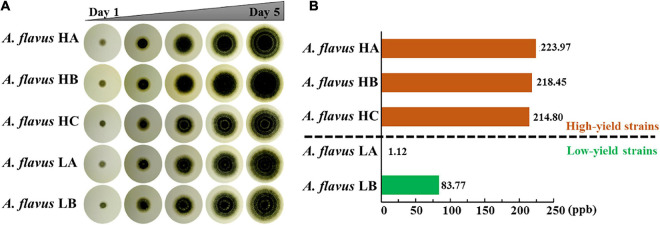
Analysis of the clone phenotype and aflatoxin production in five different *A. flavus* strains. **(A)** The clone phenotype of five different *A. flavus* strains. Colonies of the *A. flavus* HA, HB, HC, LA, and LB strains were cultured in PDA medium for 5 days. **(B)** High-performance liquid chromatography analysis of aflatoxin production in the five different *A. flavus* strains, which were cultured in PDB medium at 28°C for 5 days. The orange and green bars indicate the high- and low-aflatoxin-yield *A. flavus* strains, respectively. Each assay was performed in three biological replicates.

We obtained five different *A. flavus* strains with different AF production capabilities from the soil of peanut field across southern provinces of China, such as Hubei, Hunan and Jiangxi where the humidity and temperature are similar. The ITS sequencing and calmodulin genes sequencing results of these five *A. flavus* strains and the laboratory strain *A. flavus* NRRL3357 are shown in Supplementary Figures 1A,C. We have deposited the ITS sequences in the GenBank repository with the accession number MZ905166-MZ905170 for *A. flavus* HA, HB, HC, LA, LB, respectively. The data indicated that the five strains had high homology with *A. flavus* NRRL3357. Moreover, phylogenetic tree analysis proved that these five strains were all *A. flavus* and not any other type of fungi (Supplementary Figure 1B). To eliminate the possible effects of differences in growth rate on AF production, we continuously observed the phenotype of the five *A. flavus* isolates for 5 days. As shown in Figure 1A and Supplementary Figure 2, the diameters of these five *A. flavus* strains were almost the same, indicating that there were no conspicuous differences in their growth rate. HPLC showed that AF production in these five *A. flavus* strains was as follows: *A. flavus* HA, 223.97 ppb; *A. flavus* HB, 218.45 ppb; *A. flavus* HC, 214.80 ppb; *A. flavus* LA, 1.12 ppb; and *A. flavus* LB, 83.77 ppb (Figure 1B and Supplementary Figure 3). For further experiments, we classified these five *A. flavus* strains into two groups based on AF production using 100 ppb as the threshold (Yao et al., 2010): the high AF yield group (*A. flavus* HA, *A. flavus* HB, and *A. flavus* HC) and the low AF yield group (*A. flavus* LA and *A. flavus* LB). Although these five strains showed similar growth rates, their AF production was obviously different. Therefore, it appeared that in these five isolates of *A. flavus*, AF production was not significantly dependent on growth rate. Further analysis was thus needed to explain the differences in AF production among these five strains.

### Identification of Quantified Proteins in the Five Different *A. flavus* Strains

**FIGURE 2:**
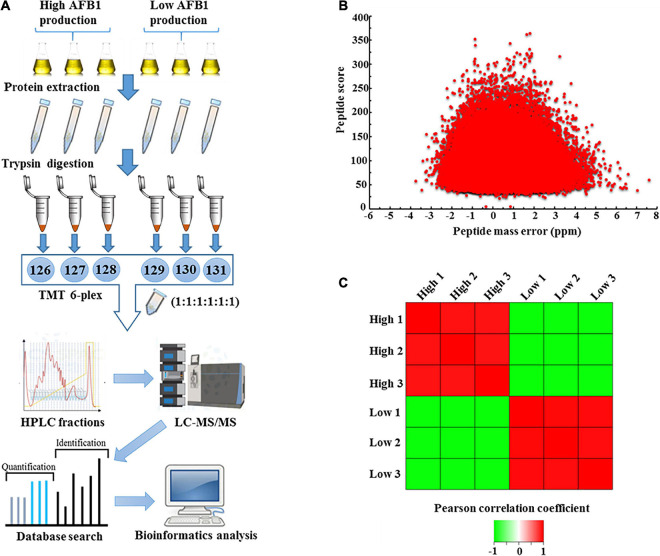
Outline of proteomic quantification in high- and low-aflatoxin-yield *A. flavus* strains. **(A)** Workflow for quantification-based proteomic analysis of *A. flavus*. **(B)** Distribution of mass error and peptide score of the quantified peptides. **(C)** Reproducibility analysis of three biological replicates based on Pearson correlation coefficients.

To evaluate the mechanism of AF production, high-yield and low-yield *A. flavus* strains were used for proteomic quantification. The experimental scheme for this study is shown in Figure 2A. Three biological replicates of high-AF-yield *A. flavus* strains (HA, HB, and HC) and low-AF-yield *A. flavus* strains (LA and LB) were mixed together individually. After protein extraction and trypsin digestion, a TMT-labeling-based proteomics approach was used for quantitation. Given that the average peptide score was 92.54 and the average absolute mass error was 1.099 ppm (Figure 2B), the quantified data appeared to be of high quality. Reproducibility analysis of three repeated biological trials showed the Pearson correlation coefficient between three high-yield aflatoxin *A. flavus* or between three low-yield aflatoxin *A. flavus* was close to 1.000, indicating that there was no significant difference between the three biological repeats. (Figure 2C). More details about all quantified proteins and biological replications were shown in Supplementary Data 1. As shown in Supplementary Figure 4A, we identified 4,862 proteins and quantified 4,363, with an estimated FDR of less than 1%. In contrast, in the wild-type and Δ*laeA* mutant strains, only 4,563 proteins were identified and 4,128 were quantified (Lv et al., 2018). Therefore, our natural isolates might provide valuable experimental materials for the studies of AF biosynthesis compared with those long-term lab cultured *A. flavus* strains, as evidenced by the 6.5 and 5.7% increase in the number of proteins identified and quantified, respectively.

### Functional Characterization of Quantified *A. flavus* Proteins

**FIGURE 3:**
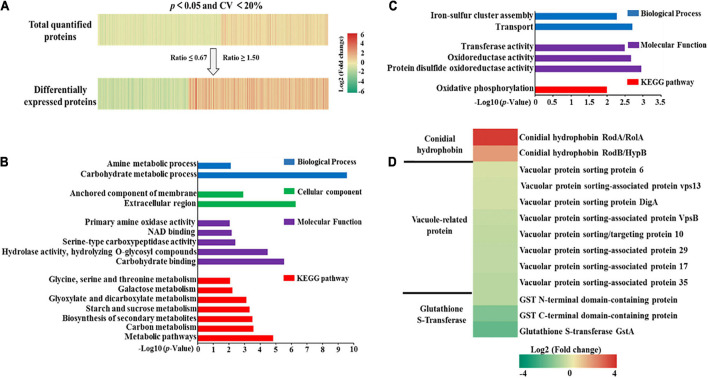
**(A)** Heatmaps of quantified proteins (top) and differentially expressed proteins (bottom). **(B)** Gene ontology and Kyoto Encyclopedia of Genes and Genomes pathway enrichment of up-regulated proteins. **(C)** Gene ontology and Kyoto Encyclopedia of Genes and Genomes pathway enrichment of down-regulated proteins. **(D)** Heatmap of some vital proteins related to aflatoxin production.

To enable a better understanding of the quantified proteins in *A. flavus*, proteins were considered differentially expressed if they exhibited a FC of ≥ 1.50 or ≤ 0.67 with a *p*-value ≤ 0.05. Unless otherwise specified, the FC represented the ratio of peak intensity in high-yield aflatoxin *A. flavus* strains compared with that in low-yield aflatoxin *A. flavus*. If FC ≥ 1.50 or ≤ 0.67, the protein was thought to be up-regulated or down-regulated, respectively. As shown in Figure 3A and Supplementary Figure 4B, among the 4,363 quantified proteins, 633 were up-regulated and 412 were down-regulated in the high-yield strains when compared with the low-yield strains. To explore the functions of these quantified DEPs, we performed GO functional analysis at level 3 across the biological process, cellular components, and molecular function modules (Supplementary Figure 5). Detailed information for these DEPs is listed in Supplementary Data 2. Among the up-regulated proteins, the top three categories related to biological process were organic substance metabolic process (239 proteins), primary metabolic process (213 proteins), and cellular metabolic process (182 proteins). Meanwhile, among the down-regulated proteins, the top three categories for biological process were cellular metabolic process (133 proteins), organic substance metabolic process (130 proteins), and primary metabolic process (118 proteins). These results suggested that both up- and down-regulated proteins were largely involved in the metabolism of certain substances, consistent with previous findings, and could have thus affected AF production (Roze et al., 2013).

We further conducted GO and pathway enrichment analyses to elucidate the potential biological functions of the quantified proteins. As shown in Figures 3B,C and Supplementary Data 2, KEGG pathway enrichment analysis indicated that the up-regulated proteins were notably enriched in carbon related metabolism (*p* = 2.64E-4), while the down-regulated proteins were slightly enriched in oxidative phosphorylation (*p* = 1.00E-2). Further, no proteins were enriched in the cellular components module. These results proved that these up-regulated proteins involving in carbon metabolism had a direct relationship with aflatoxin production based on the fact that aflatoxin was secondary metabolites regulated by TCA cycle, glycolysis and pentose phosphate pathway (Gupta et al., 1977; Buchanan and Lewis, 1984; Yan et al., 2012). As far as these down-regulated proteins, we guess these proteins might regulate the aflatoxin production through oxidative stress responses as the fact that oxidative phosphorylation was central to *A. flavus* oxidative stress responses (Fountain et al., 2019).

When we ranked the DEPs according to the ratio of concentration between high- and low-AF-yield *A. flavus* strains, we found that the conidial hydrophobin proteins RodA (B8NTJ8) and RodB (B8N5T3) were significantly up-regulated, with a FC of 14.05 and 3.88, respectively (Figure 3D). It has been reported that vacuole-associated proteins can promote AF synthesis in fungi (Yang et al., 2021). However, in the present study, we quantified eight vacuole-associated proteins and found that their FC ranged between 0.67 and 1.50, indicating that they show no significant differences under natural conditions. In contrast, when we examined the expression of three glutathione S-transferase (GST) proteins, which have been reported to correlate with AF production (Ziglari et al., 2008), we observed that GST C-terminal domain-containing protein (B8NBY8) and glutathione S-transferase GstA (B8N3U8) were down-regulated. This further validated our domain enrichment analysis, shown in Supplementary Figure 6.

### Quantified Proteins Involved in Carbon-Related Metabolism

**FIGURE 4:**
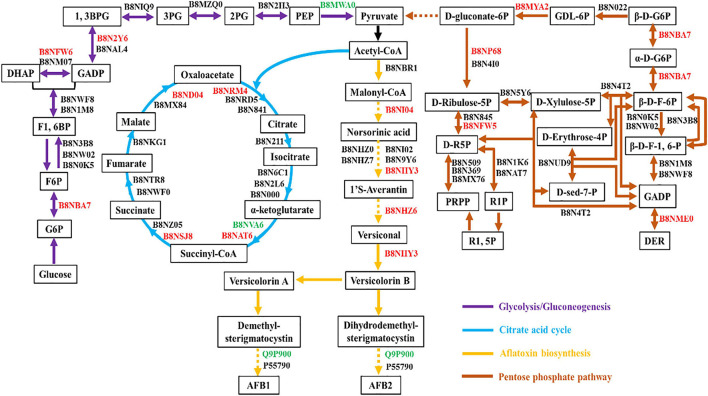
Carbohydrate metabolic pathway annotation for quantified proteins. Glycolysis, the citric acid cycle, aflatoxin biosynthesis, and the pentose phosphate pathway are represented with purple, blue, yellow, and brown lines, respectively. All quantified proteins are marked in black [no significant change (0.67<FC<1.50)], red [up-regulated (FC ≥ 1.50)], or green [down-regulated (FC ≤ 0.67)] according to the fold change between high- and low-yield *A. flavus* strains.

To elucidate the relationship between AF production and material metabolism, pathway annotation for quantified proteins was performed based on the KEGG database. Acetyl-CoA is the primary substrate for AF synthesis (Caceres et al., 2020). Hence, the main metabolic pathways involving acetyl-CoA, including glycolysis, the citric acid cycle, AF biosynthesis, and the pentose phosphate pathway were analyzed. As shown in Figure 4, fourteen enzymes were involved in glycolysis/gluconeogenesis, 16 in the citric acid cycle, 13 in AF biosynthesis, and 23 in the pentose phosphate pathway. Most of these quantified proteins were significantly up-regulated in high AF yield strains. The glucose-6-phosphate isomerase (B8NBA7) involved in glycolysis and pentose phosphate pathway was up-regulated 2.17 fold. Triosephosphate isomerase (B8NFW6) and glyceraldehyde-3-phosphate dehydrogenase (B8N2Y6), both involved in glycolysis, showed 1.67- and 1.85-fold up-regulation, respectively. Four enzymes involved in the citric acid cycle, including citrate synthase (B8NRM4, FC = 1.51), dihydrolipoyl dehydrogenase (B8NAT6, FC = 1.78), succinate-CoA ligase (B8NSJ8, FC = 1.54), and malate dehydrogenase (B8ND04, FC = 2.25), were up-regulated. These up-regulated proteins have previously been identified in proteomic research (Ren et al., 2018; Wang et al., 2019). The vital enzymes involved in AF biosynthesis, including aflC (B8NI04, FC = 1.94), aflK (B8NHY3, FC = 2.03), and aflJ (B8NHZ6, FC = 1.56), were also found to be up-regulated. In the pentose phosphate pathway, except for glucose-6-phosphate isomerase (B8NBA7), the other four enzymes 6-phosphogluconolactonase (B8MYA2), 6-phosphogluconate dehydrogenase (B8NP68), ribose/galactose isomerase (B8NFW5), and 2-deoxy-D-ribose 5-phosphate aldolase (B8NME0) showed a significant increase, with 1. 56-, 2. 11-, 4. 12-, and 1.75-fold up-regulation. Except for ribose/galactose isomerase (B8NFW5), these proteins have also been identified in other proteomics studies (Ren et al., 2018). In contrast, three enzymes, including pyruvate kinase (B8MWA0, FC = 0.64), dihydrolipoyllysine succinyltransferase (B8NVA6, FC = 0.62), and demethylsterigmatocystin 6-*O*-methyltransferase (Q9P900, FC = 0.63), were found to be down-regulated, although the FC was not very significant. In addition, we have investigated the global regulators involved in AF biosynthesis according to the latest review article (Caceres et al., 2020). Unfortunately, we did not find significant differences between high- and low-AF-yield *A. flavus* strains for the identified global regulator (B8NSN6, 1.36) or even could not identify those global regulators (Supplementary Table 2), probably due to their low expression in natural isolates of *A. flavus*. Together, our results suggested that these DEPs might play an important role in the modulation of AF biosynthesis in natural isolates of *A. flavus* at multiple levels.

### Protein–Protein Interaction Network of Quantified Proteins

**FIGURE 5:**
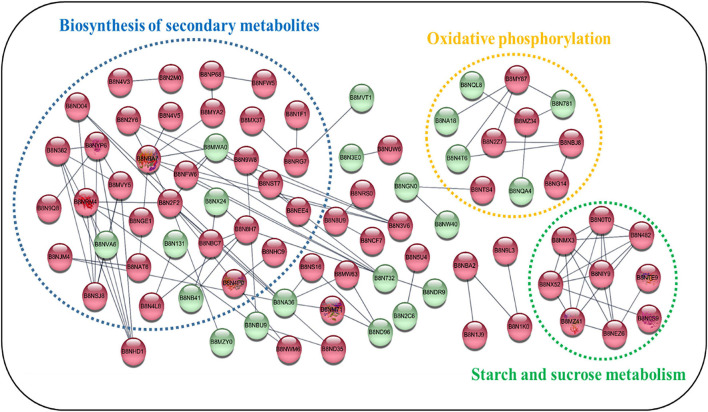
Predicted protein–protein interactions among differentially expressed proteins (DEPs) across metabolic pathways. The STRING database was used to examine proteins that showed increased or decreased expression in *A. flavus* isolated from natural environments. Each node in the network represents a DEP. The red and green nodes represent up- and down-regulated proteins, respectively. Clusters of interest are indicated by colored labels. The proteins involved in the biosynthesis of secondary metabolites, oxidative phosphorylation, and starch and sucrose metabolism are circled in dotted ellipses.

To examine the interactions between the DEPs detected in the examined isolates, we used the STRING database to search for potential physical, co-expression, and co-occurrence interactions (Szklarczyk et al., 2015). As shown in Figure 5, proteins with significant changes were selected for PPI analysis. To improve the confidence of the interaction network, only experimental evidence was considered. Overall, three smaller interaction groups were identified, and these consisted of secondary biosynthesis metabolites and components of the oxidative phosphorylation, starch and sucrose metabolism pathways. Several hub proteins exhibiting physical and co-expression interactions with multiple proteins across diverse pathways were identified. For example, malate dehydrogenase (B8ND04), a citric acid cycle enzyme, controlled the production of secondary metabolites. Phosphoenolpyruvate carboxykinase (B8N2F2), which is involved in gluconeogenesis, regulated the synthesis of ATP. Among the down-regulated proteins, cytochrome c oxidase subunit Va (B8NQA4) and cytochrome C1/Cyt1 (B8MYK0) regulated oxidative phosphorylation through cytochromes. These results indicated that the down-regulated proteins may control AF production *via* oxidative phosphorylation, consistent with previous research (Tian et al., 2020). With regard to starch and sucrose metabolism, there were nine proteins showing significant up-regulation, but no down-regulated proteins were observed in this cluster. Domain enrichment analysis (Supplementary Figure 6) also showed that up-regulated proteins were mostly glycoside hydrolases (*p* = 2.71E-8), which catalyze the hydrolysis of the glycosidic linkage in glycosides and provide more energy. We postulated that natural isolates of *A. flavus* need more starch and sucrose metabolism-related enzymes to obtain energy and produce AF. Kernel amylase (B8NMX3) has previously been found to play a role in the induction of AF biosynthesis (Woloshuk et al., 1997). Our findings are consistent with previous research showing that starch and sucrose metabolism is positively correlated with AF production (Liu et al., 2016).

### High-Resolution Multiple Reaction Monitoring Validation for Tandem Mass Tag Quantification

**FIGURE 6:**
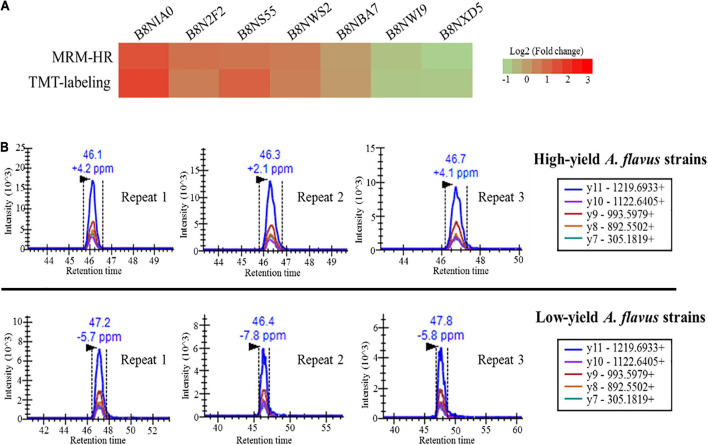
High-resolution multiple reaction monitoring (MRM-HR) verification of candidate proteins. **(A)** Heatmap showing the ratio of nine peptides from relevant candidate proteins obtained using MRM-HR and the TMT-labeling method. **(B)** Representative MRM-HR chromatograms of the peptide DVGIVGLPVTWDR from glucose-6-phosphate isomerase (B8NBA7). Quantification analysis using high-performance liquid chromatography coupled with tandem mass spectrometry. Chromatograms were obtained in the MRM-HR mode. The upper and lower three chromatograms represent the three biological replicates for high- and low-aflatoxin-yield *A. flavus* strains, respectively.

High-resolution multiple reaction monitoring is the gold standard for candidate verification of quantified proteins (Peterson et al., 2012; Schilling et al., 2015). Targeted MRM proteomics is considered a better protein quantification method than western blotting (Prasad, 2014). Herein, to verify the reliability of TMT-labeling-based protein quantification, 23 quantified proteins were subjected to MRM-HR verification according to the FC, score, coverage, *p*-value, and biological functions obtained from the TMT-labeling method. The candidate proteins are listed in Supplementary Data 3. Seventeen proteins were successfully identified and quantified using MRM-HR, among which seven showed the same tendency (up- or down-regulation) observed using the TMT-labeling method. We further compared the peptides of candidate proteins between MRM-HR and the TMT-labeling method. As shown in Supplementary Table 3, the ratios quantified using MRM-HR were highly consistent with those obtained using the TMT-labeling method. The MRM-HR spectra of the glucose-6-phosphate isomerase (B8NBA7) peptide DVGIVGLPVTWDR is shown in Figure 6, and six other spectra from relevant peptides are shown in Supplementary Figure 7. These results indicated that TMT-labeling-based proteome quantification exhibited good consistency with MRM-HR findings. Our quantification had high credibility and could provide more authentic information for further research on AF biosynthesis in *A. flavus* growing in ecological niches.

## Discussion

Although many efforts have been made to study the mechanisms underlying AF biosynthesis using different culture conditions, little attention has been paid to these mechanisms in natural isolates of *A. flavus*. We hypothesize that the strains isolated from the similar environment could be good experimental materials to study the proteomics involving Aflatoxin biosynthesis because these strains are scarcely influenced by various culturing conditions during long-term culture or transfer between different labs. In this study, we aimed to elucidate the differences in protein expression across natural isolates of *A. flavus* showing different AF yields under the same field conditions. Importantly, we could utilize these results to gain novel insights into AF production.

Through our quantitative proteomic profiling of *A. flavus* strains isolated from natural environments, we identified 4,862 proteins and quantified 4,363 (Supplementary Figure 4), of which 633 were up-regulated and 412 were down-regulated in the high-yield *A. flavus* strains. We noted that the up-regulated proteins in the high AF yield group were mostly enriched in carbon-related metabolism (*p* = 2.64E-4), while the down-regulated proteins were enriched in oxidative phosphorylation (*p* = 1.00E-2) (Figure 3). Given that AF is a secondary metabolite regulated by the citric acid cycle, glycolysis, and pentose phosphate pathway, these results proved that the up-regulated proteins involved in carbon-related metabolism had a direct relationship with AF production (Gupta et al., 1977; Buchanan and Lewis, 1984; Yan et al., 2012). Notably, we showed for the first time that proteins enriched in oxidative phosphorylation are down-regulated in *A. flavus* strains obtained from natural ecological environments. We believe that these proteins may regulate AF production through oxidative stress responses because oxidative phosphorylation is central to the *A. flavus* oxidative stress responses (Fountain et al., 2019), although more experiments are necessary to prove this supposition.

When we ranked DEPs according to the FC between high- and low-yield *A. flavus* strains, we obtained some new insights. We found that the conidial hydrophobin proteins RodA and RodB were significantly up-regulated, with a FC of 14.05 and 3.88, respectively. Hydrophobins play a role in the interaction between fungi and their environment and in the attachment of fungi to solid supports such as crops (Linder et al., 2005), However, little is known about their function in AF biosynthesis. We postulated that these up-regulated hydrophobins could help the *A*. *flavus* strains isolated from natural environments invade crops and thereafter produce more AFs (Chang et al., 2012; Yang et al., 2020). In addition, it has been reported that vacuole-associated proteins can promote AF synthesis in fungi and also promote the consequent export of AFs by regulating vacuolar homeostasis (Chanda et al., 2009; Yang et al., 2021). In our study, we quantified eight vacuole-associated proteins and found that they showed FCs between 0.67 and 1.50. This could be because *A. flavus* strains require vacuole-associated proteins to transport AFs in a liquid medium, while these proteins are not necessary when *A. flavus* strains grow on natural solid substrates such as maize or peanuts (Yang et al., 2021). Interestingly, Ziglari et al. (2008) found that the mean GST concentration was about 2.9-fold higher in AF-producing fungi than in non-toxigenic isolates. However, in our study, GST proteins showed significant down-regulation in high-AF-yield strains when compared with low-yield strains. We know that the GST superfamily is composed of a set of enzymes involved in detoxification (Roncalli et al., 2015). This conflicting finding may have been obtained because GST concentration was examined using the enzyme-linked immunosorbent assay in previous studies, providing the concentration of total GST, while our proteomics data quantified single GST proteins. Moreover, GST proteins have been found to eliminate toxic free radicals, as is observed for AFs (Ziglari and Allameh, 2013). Hence, low-yield *A. flavus* strains may need more GST proteins to reduce toxic free radicals under natural conditions. More experiments are needed to confirm this speculation.

We also analyzed the DEPs regulating the flux of acetyl CoA, which is the initial substrate for AF biosynthesis. Of the DEPs, 14 were enzymes involved in glycolysis/gluconeogenesis, 16 were involved in the citric acid cycle, 13 were involved in AF biosynthesis, and 23 were involved in the pentose phosphate pathway. Among these quantified proteins, most were significantly up-regulated, implying that they may regulate AF production at multiple levels. Moreover, the vital enzymes involved in AF biosynthesis, including aflC, aflK, and aflJ, were also found to be significantly up-regulated. These results were consistent with previous findings obtained from gene deletion, complementation, and RNA interference experiments that showed that aflC, aflK, and aflJ all exert a positive effect on AF production (Du et al., 2007; Ren et al., 2017; Thakare et al., 2017). Although many of these up-regulated proteins had been identified in previous proteomics studies, three enzymes observed to be down-regulated in the present study—pyruvate kinase, dihydrolipoyllysine succinyltransferase, and demethylsterigmatocystin 6-*O*-methyltransferase—were found to be up-regulated in *A. flavus* strains cultured in artificial conditions (Flipphi et al., 2009; Jamali et al., 2013; Ren et al., 2016). This could be because different growth conditions may affect protein patterns of *A. flavus*. The global regulators involved in AF biosynthesis have also been analyzed. However, we could not identify these global regulators or their expression has no significant difference between high- and low-yield strains due to their low expression. PPI analysis of DEPs revealed three enriched clusters that included secondary biosynthesis metabolites and oxidative phosphorylation, starch, and sucrose metabolism components. Several hub proteins exhibiting physical and co-expression interactions with multiple proteins across diverse pathways were also identified, including malate dehydrogenase, phosphoenolpyruvate carboxykinase, cytochrome c oxidase subunit Va, and cytochrome C1/Cyt1. Additionally, all of the nine proteins enriched in starch and sucrose metabolism were found to be significantly up-regulated, indicating that the high-yield *A. flavus* strains needed more energy to produce AFs under natural conditions. Further experimental interventions are required to confirm the above hypotheses and elucidate the potential regulatory mechanism of these DEPs in *A. flavus* strains isolated from natural environments.

## Conclusion

We performed a comprehensive quantification-based proteomic analysis of *A. flavus* strains isolated from natural environments showing high and low AF production capabilities. The proteomic quantification was performed using the TMT-labeling method and validated using the MRM-HR method. In total, we quantified 4,363 proteins in natural isolates of *A. flavus*, among which 1,045 were DEPs. Bioinformatics analysis indicated that these proteins were mainly enriched in carbon-related pathways, as observed in laboratory *A. flavus* strains. However, GST proteins were significantly down-regulated in high-yield *A. flavus* strains, which contradicted a previous study conducted in *A. flavus* strains under artificial conditions (Saxena et al., 1989). Overall, our study provides a new strategy for studying the underlying mechanism associated with AF biosynthesis. Further, our results can assist future studies on fungal growth and mycotoxin production in natural environments. Moreover, through the proteomic profiling of natural *A. flavus* isolates showing high and low AF yield, we provide novel insights into AF production. Our findings could help in the development of innovative strategies for controlling toxin biosynthesis in food and other agricultural products. Aflatoxin biosynthesis is a complex process, involving the regulation of many intermediate products. Multiomics analysis involving transcriptomics, metabolomics and proteomics could further facilitate a deep understanding of the AFs biosynthesis mechanism.

## Data Availability Statement

The datasets presented in this study can be found in online repositories. The names of the repository/repositories and accession number(s) can be found below: ProteomeXchange dataset PXD027517.

## Author Contributions

TL finished the article experiments, data analysis, and wrote the manuscript. ZZ provided the natural isolates of *Aspergillus flavus* strains. YW helped to finish the phenotype experiments. RH and YY conducted the MS data acquisition, and provided the idea and experiments support. YL, JZ, and ML helped to finish the experiments and revised the article. All authors contributed to the article and approved the submitted version.

## Conflict of Interest

The authors declare that the research was conducted in the absence of any commercial or financial relationships that could be construed as a potential conflict of interest. The reviewer FX declared a shared affiliation with one of the authors, ZZ, to the handling editor at time of review.

## Publisher’s Note

All claims expressed in this article are solely those of the authors and do not necessarily represent those of their affiliated organizations, or those of the publisher, the editors and the reviewers. Any product that may be evaluated in this article, or claim that may be made by its manufacturer, is not guaranteed or endorsed by the publisher.

## Footnotes

1
https://www.kegg.jp/
