# Supplementary material for: Quantitative Proteomic Analysis for High- and Low-Aflatoxin-Yield Aspergillus flavus Strains Isolated From Natural Environments
Source: Front Microbiol. 2021 Sep 21;12:741875. doi: 10.3389/fmicb.2021.741875 (PMC8491651; doi:10.3389/fmicb.2021.741875)
Supplement: Supplementary file 4 [file Data_Sheet_4.docx]

**Quantitative proteomic analysis for high- and low-aflatoxin-yield *Aspergillus flavus* strains isolated from natural environments**

Tao Li^1,2,#^, Zhaowei Zhang^3,#^, Yu Wang^1^, Ying Li^1,2^, Jiang Zhu^1,2^, Rui Hu^1,2,^*, Yunhuang Yang^1,2,^*, Maili Liu^1,2^

^1^State Key Laboratory of Magnetic Resonance and Atomic Molecular Physics, Key Laboratory of Magnetic Resonance in Biological Systems, National Center for Magnetic Resonance in Wuhan, Wuhan Institute of Physics and Mathematics, Innovation Academy for Precision Measurement Science and Technology, Chinese Academy of Sciences – Wuhan National Laboratory for Optoelectronics, Huazhong University of Science and Technology, Wuhan, 430071, China;

^2^ University of Chinese Academy of Sciences*,* Beijing 100049, China;

^3^ Chinese Academy of Agricultural Sciences, Wuhan 430062, China;

# These authors contributed equally to this work.

* Corresponding author:

Email: hurui@apm.ac.cn; yang_yh@apm.ac.cn.

**Table S1.** TMT Labeling information

| Sample Groups | TMT Label |
| --- | --- |
| Low-aflatoxin 1 | 126 |
| Low-aflatoxin 2 | 127 |
| Low-aflatoxin 3 | 128 |
| High-aflatoxin 1 | 129 |
| High-aflatoxin 2 | 130 |
| High-aflatoxin 3 | 131 |

**Table S2. The global regulators involved in AF biosynthesis**

| Protein name | Uniprot ID | Ratio (High/Low) | References |
| --- | --- | --- | --- |
| AflR | P41765 | Not found | Applied and environmental microbiology, 1994, 60, 2408-2414 |
| AflS | B8NHZ8 | Not found | Molecular genetics and genomics, 2003, 268,711-719 |
| LaeA | B8N406 | Not found | Science 2008, 320, 1504–1506 |
| VeA | B8NIF0 | Not found | Fungal genetics and biology, 2008, 45, 1053-1061 |
| AreA | B8NSN6 | 1.36 | Toxins, 2019, 11, 718 |
| Hbx1 | A0A364LVL0 | Not found | G3: Genes\| Genomes\| Genetics, 2019, 9, 167-178 |
| MsnA | A0A364M5V9 | Not found | Toxins, 2011, 3, 82-104 |

**Table S3**. MRM-HR verification for TMT-labeling quantification

| Uniprot ID | Peptide Sequence | TMT-quantified | MRM-HR |
| --- | --- | --- | --- |
|  |  | High/Low ratio | High/Low ratio |
| B8NIA0 | YTDYDGPSIR | 2.48 | 2.86 |
| B8N2F2 | TTLSADPNR | 1.79 | 1.57 |
| B8NS55 | GFQPDDQK | 1.73 | 2.11 |
| B8NWS2 | TFDPYVIPLLEK | 1.59 | 1.55 |
| B8NBA7 | DSNPETTLFLIASK | 1.14 | 1.16 |
| B8NWI9 | AMEGLVAAGAVPK | 0.77 | 0.74 |
| B8NXD5 | SELEPQLPEGSDGK | 0.66 | 0.73 |


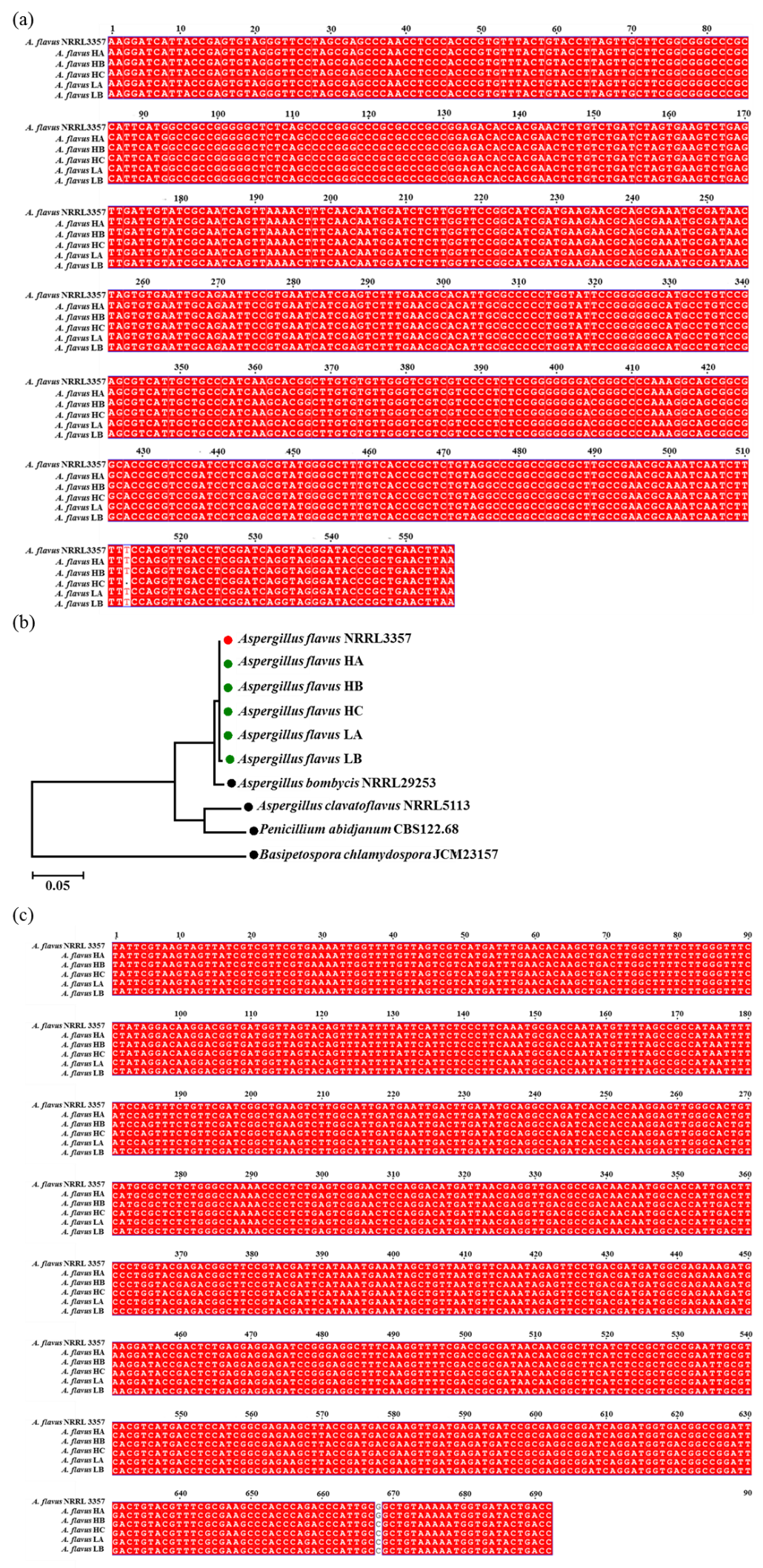


**Figure S1. Five *A. flavus* strains isolated from natural conditions were identified by amplification and sequencing of the ITS region.** (a) ITS sequence alignment of five *A. flavus* strains with *A. flavus* NRRL3357. (b) Phylogenetic tree analysis of five isolated *A. flavus* strains compared with *A. flavus* NRRL3357 and other fungus based on ITS sequence alignment. (c) Calmodulin genes sequence alignment of five natural *A. flavus* strains with *A. flavus* NRRL3357.


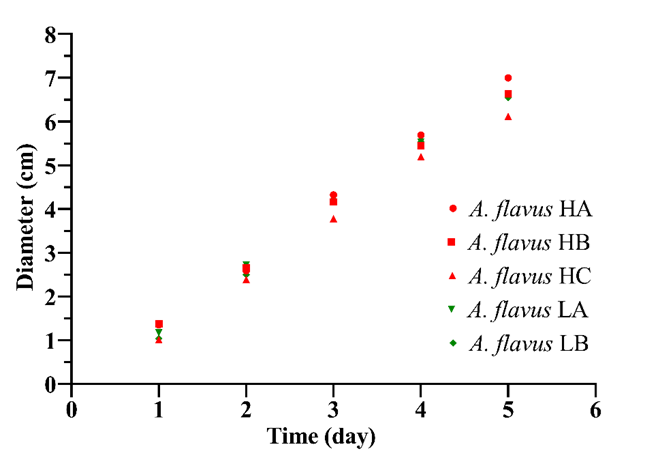


**Figure S2. The Diameter of clones from five different *A. flavus* strains.** The diameter of these five *A. flavus* strains was evaluated every 24 hours.


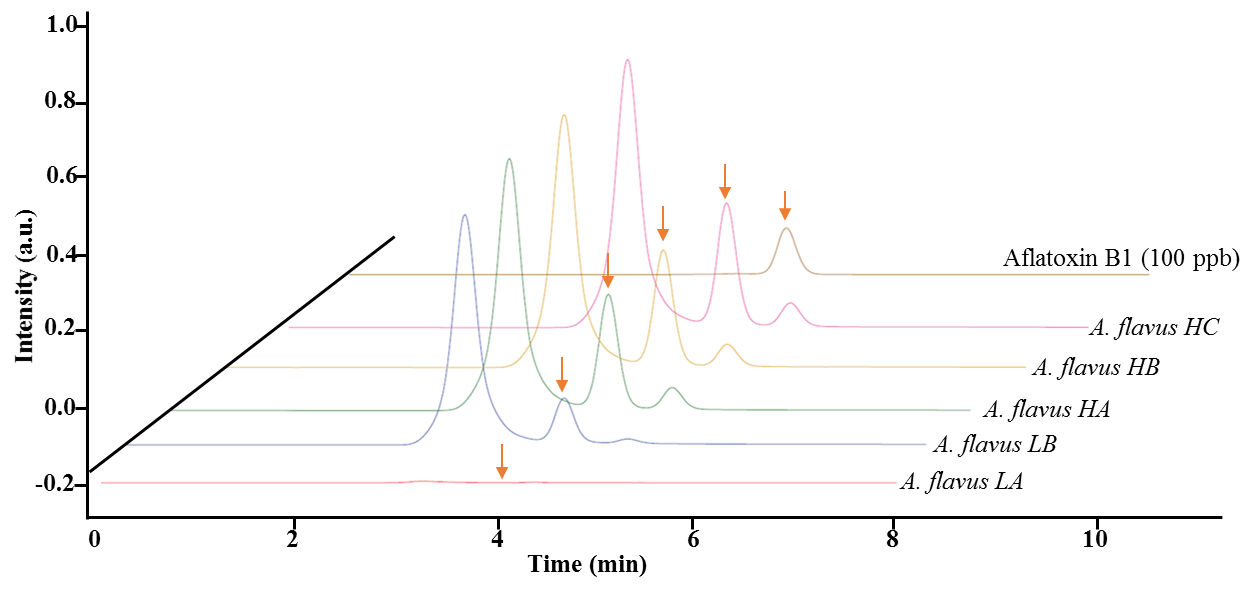


**Figure S3. HPLC analysis of aflatoxin production.** *A. flavus* LA, LB, HA, HB and LC strains were inoculated in PDB liquid media with the same concentration at 28 for 5days. Aflatoxin was extracted using a previously described protocol ([Wacoo et al., 2014](#_ENREF_2)). AFB1 (100ppb) was used as standard for quantification analysis. The red arrow stood for the peak of AFB1. To eliminate the random errors caused by the system, each *A. flavus* strains had three biological repeats and were mixed together for further aflatoxin production analysis.


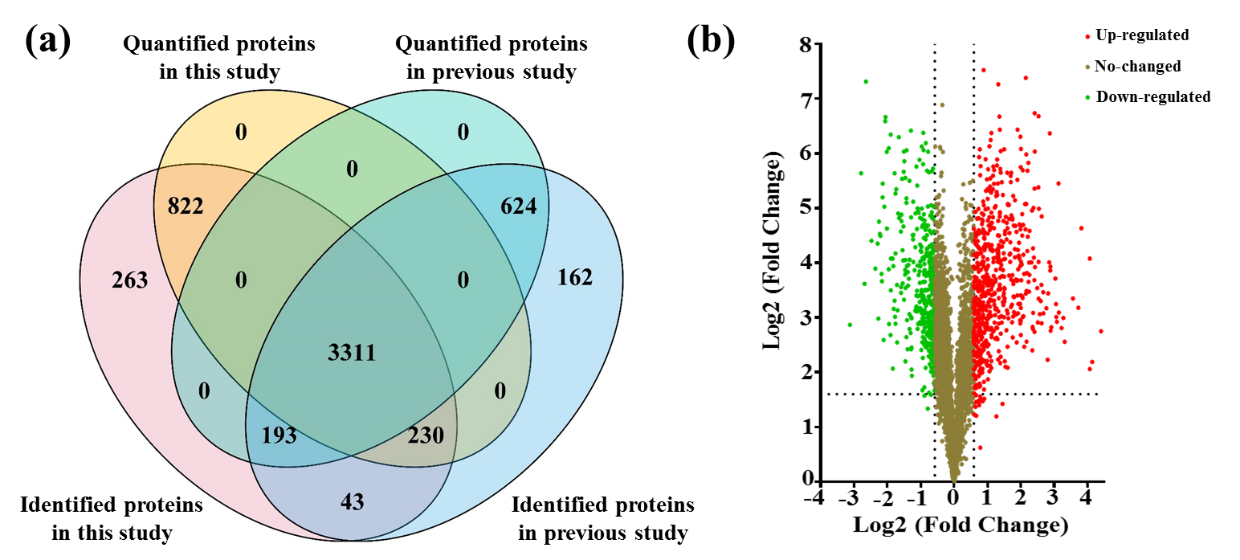


**Figure S4. The Venn plot and Volcanic plot of identified and quantified proteins.** (a) The Venn plot of all identified and quantified proteins. The previous research was the quantitative proteomics conducted by Lv *et al* with TMT-labeling method ([Lv et al., 2018](#_ENREF_1)). (b) The Volcanic plot of quantified proteins. The cross virtual line represented the threshold of p-value=0.05 and the vertical virtual line represented the threshold of Fold Change1.50 or 0.67, respectively.


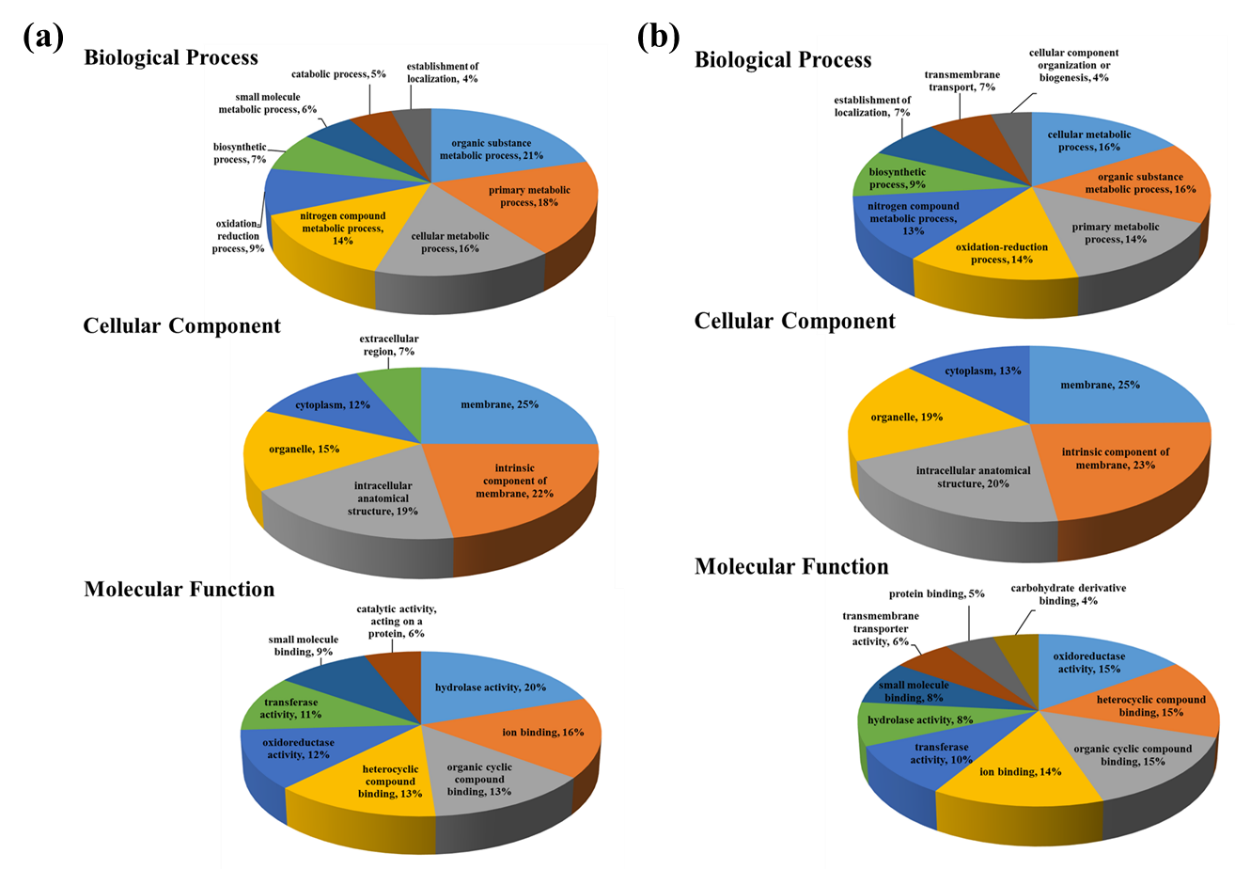


**Figure S5. Gene Ontology analysis of differentially expressed quantified proteins based on TMT-labeling method.** (a) Biological process, cellular component and molecular function analysis of up-regulated proteins based on GO terms level 3. (b) Biological process, cellular component and molecular function analysis of down-regulated proteins based on GO terms level 3.


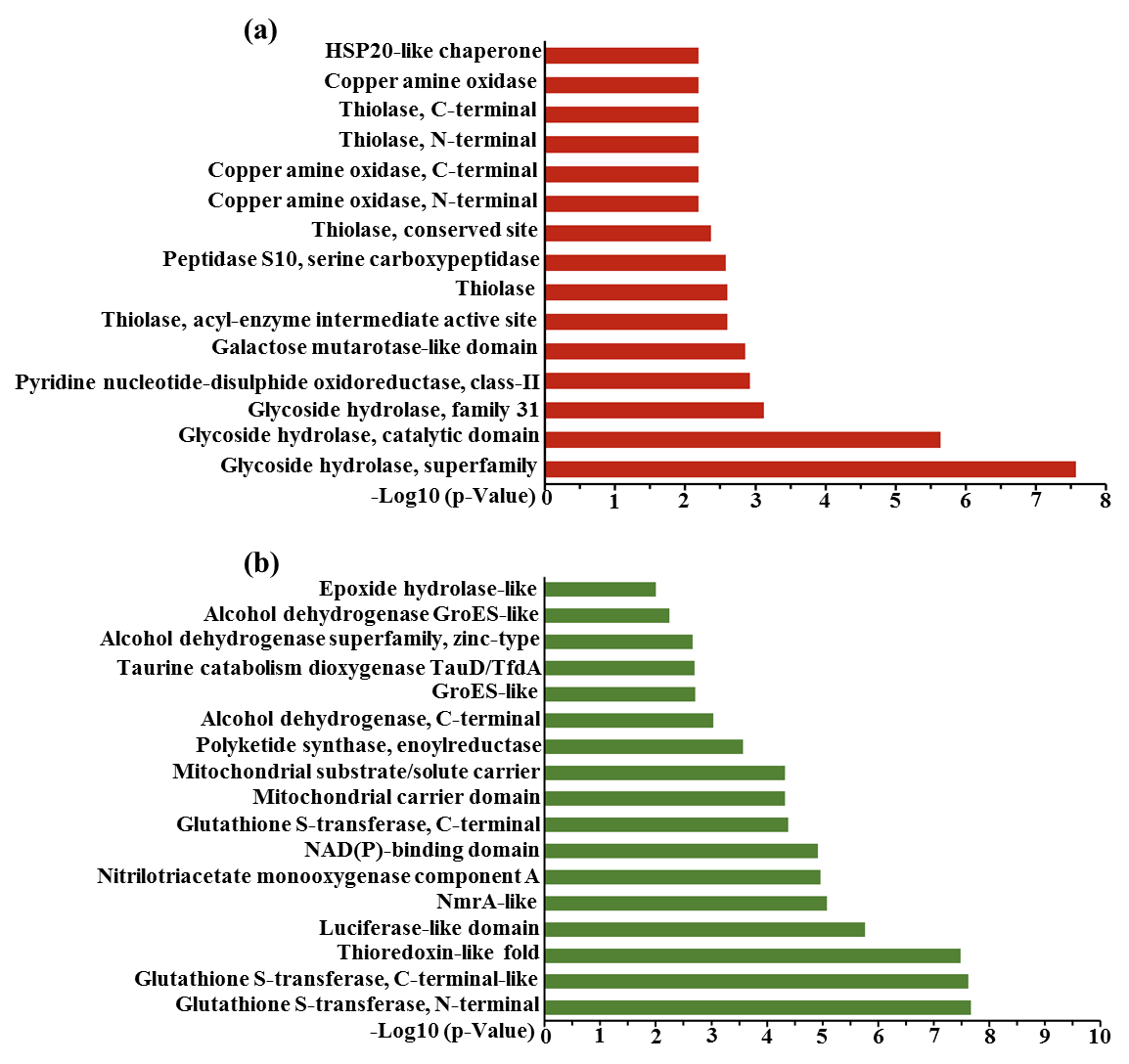


**Figure S6. Domain enrichment analysis for the up-regulated and down-regulated proteins.** The red and green bar represented the upregulated and downregulated proteins respectively. A *p*-value less than 0.01 (typically≤0.01) is statistically significant.


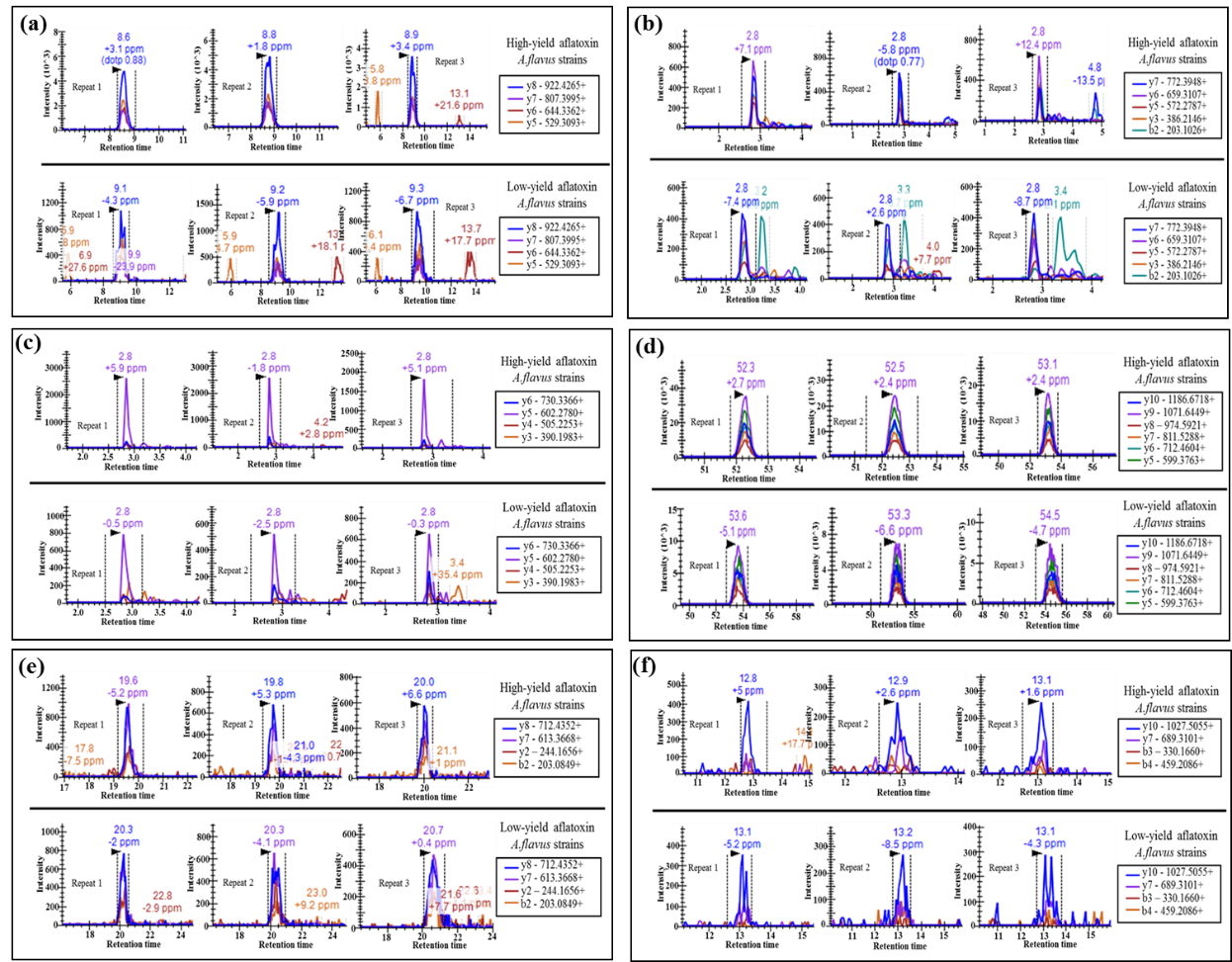


**Figure S7. MRM-HR verification of candidate proteins from TMT-labeled methods.** The representative MRM-HR chromatograms of eight peptides from candidate protein. Quantification analysis by high-performance liquid chromatography to tandem mass spectrometry (HPLC-MS/MS). Chromatograms were obtained in high-resolution multiple reaction monitoring (MRM-HR) mode. (a)-(f) stands for the three biological repeats result from B8NIA0, B8NBA7, B8N2F2, B8NWS2, B8NWI9 and B8NXD5 respectively. The peptide sequence of candidate proteins was listed in Table S2.

**References**

Lv, Y., Lv, A., Zhai, H., Zhang, S., Li, L., Cai, J., and Hu, Y. (2018). Insight into the global regulation of laeA in Aspergillus flavus based on proteomic profiling. *Int J Food Microbiol* 284**,** 11-21.

Wacoo, A.P., Wendiro, D., Vuzi, P.C., and Hawumba, J.F. (2014). Methods for Detection of Aflatoxins in Agricultural Food Crops. *Journal of Applied Chemistry* 2014**,** 1-15.
